# The Ross Procedure in a Case of Baraitser-Winter Syndrome: A Case Report

**DOI:** 10.7759/cureus.52331

**Published:** 2024-01-15

**Authors:** Raghad A Alotabi, Orjowan Z Alamri, Shahad E Alenezi, Ihab Suliman

**Affiliations:** 1 Medicine, King Saud Bin Abdulaziz University for Health Sciences College of Medicine, Riyadh, SAU; 2 Cardiology, King Abdulaziz Medical City, King Abdulaziz Cardiac Center, Ministry of National Guard Health Affairs, Riyadh, SAU

**Keywords:** baraitser-winter syndrome, prolapsed mitral valve, aortic valve insufficiency, bicuspid aortic valve, ross procedure

## Abstract

Baraitser-Winter syndrome (BRWS) is a rare genetic disorder caused by mutations in the ACTB and ACTG1 genes. It is characterized by intellectual disability, physical malformations, and dysmorphic craniofacial features. Additionally, cardiovascular abnormalities may also be present. We present a case of a 15-year-old boy with BRWS associated with congenital bicuspid aortic valve and severe aortic insufficiency which was managed successfully with Ross procedure.

## Introduction

Baraitser-Winter syndrome (BRWS) is a rare autosomal dominant disorder, first described by Michael Baraitser and Robin Winter in 1988 [1]. This congenital anomaly syndrome encompasses a range of manifestations, including intellectual disability, varying dysmorphic features, and multi-organ malformations. Due to its infrequency, the exact prevalence is not firmly established, with fewer than 100 cases documented in medical literature [2]. A gain-of-function mutation in ACTB or ACTG genes encoding actin is implicated in causing this rare genetic condition [3]. Although the phenotypic expression varies greatly, the most severe instances of the syndrome are associated with two specific pathogenic variants: mutation c.359C>T; p.(Thr120Ile) in the ACTB gene [4,5] and mutation c.608C>T; p.Thr203Met in the ACTG1 gene [6,7].

As of now, there are no established clinical diagnostic criteria for BRWS, but it should be suspected in individuals displaying a suggestive facial phenotype paired with a confirmed pathogenic variant in either ACTB or ACTG1 through molecular genetic testing [8]. Some of the distinctive craniofacial characteristics include prominent metopic ridging or trigonocephaly, widely spaced eyes, bilateral ptosis, highly arched eyebrows, ocular coloboma, small ears, and wide, short, thick, and upturned nose with large tip. BRWS also exhibits correlations with brain anomalies, specifically pachygyria (frontal or predominantly central), subcortical band heterotopia, corpus callosum abnormality, and often, epilepsy of variable severity. The degree of intellectual disability ranges from significant in individuals with lissencephaly to mild intellectual disability in some individuals with anterior pachygyria [9]. Additionally, other reported anomalies include cardiovascular malformations such as patent ductus arteriosus, ventricular or atrial septal defects, abnormal aortic valve, aortic stenosis, mitral valve regurgitation, and tricuspid regurgitation [8].

## Case presentation

This case pertains to a 15-year-old male who presented with multiple congenital cardiac anomalies and received a diagnosis of BRWS at the age of seven. The patient exhibits phenotypic features consistent with BRWS, including hypertelorism, protruded tongue, wide nasal bridge, low-set ears, low posterior hairline with neck webbing and axillary pterygium. Additionally, the patient demonstrates an intellectual disability, evidenced by difficulty reading and limited verbal communication skills. At the age of six, Noonan syndrome was initially considered due to cardiac anomalies and suggestive dysmorphic features. However, Noonan syndrome was ruled out after the genetic testing for PTPN11, RAF1, SOS1, and SHOC2 yielded no pathogenic variants, and chromosomal analysis revealed a normal karyotype. Subsequently, the patient was diagnosed with BRWS based on clinical phenotype and the identification of a heterozygous pathogenic variant in the ACTG1 gene (c.50G>A; p.C17Y) through whole exome sequencing.

Since birth, the patient has presented with multiple medical issues, including congenital bicuspid aortic valve with severe aortic valve insufficiency, myxomatous mitral valve with prolapse of anterior and posterior leaflets, and mild mitral regurgitation. Furthermore, the patient has experienced delayed developmental milestones, a horseshoe kidney, and a complete corpus callosum with thinning of its distal part. Given the complexity of the patient's condition, a multidisciplinary team consisting of a pediatrician, genetics specialist, urologist, and cardiologist has been involved in the patient's care.

**Figure 1:**
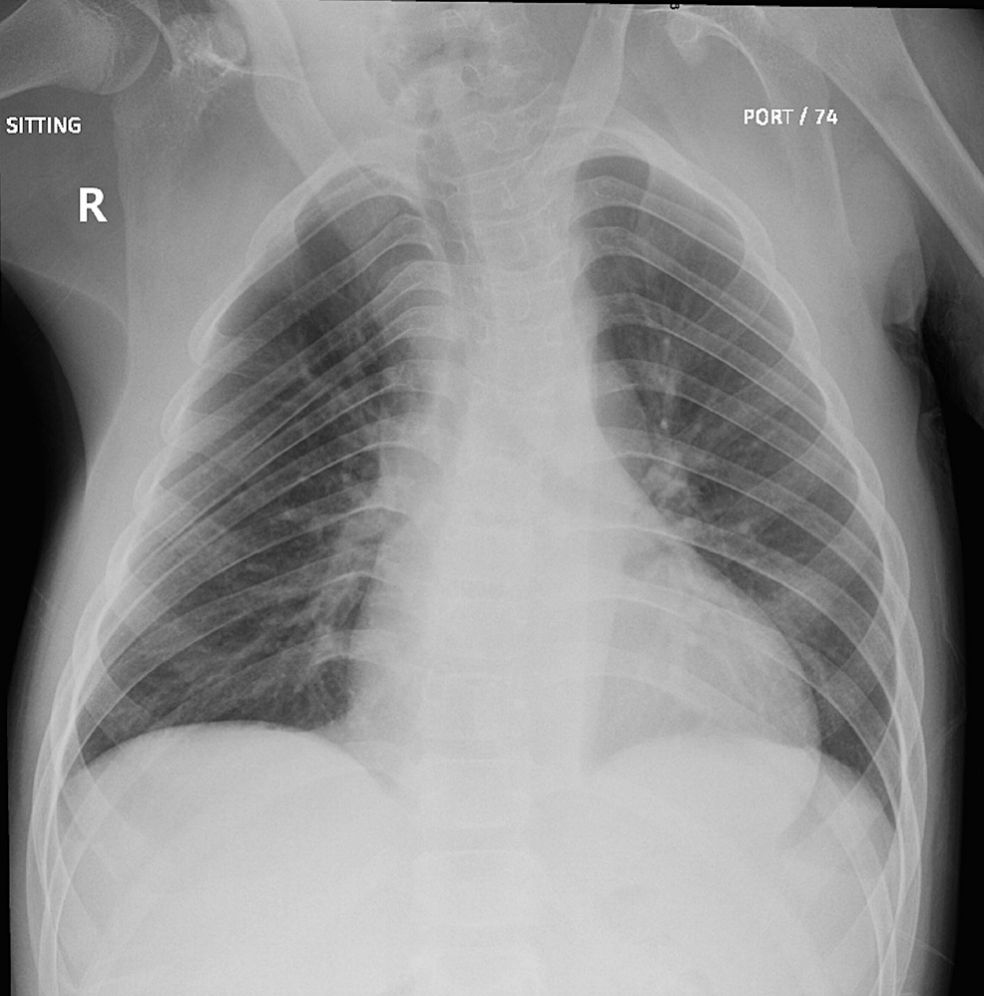
Pre-op Chest X-ray

**Figure 2:**
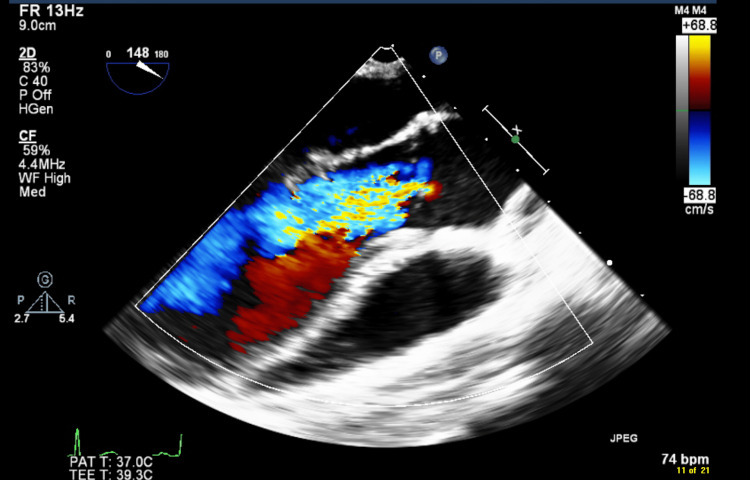
Aortic Valve

The patient underwent regular follow-ups at the cardiology outpatient department and was well-controlled and asymptomatic while on lisinopril until the age of 13, when he began experiencing progressive shortness of breath and fatigue during exertion. This deterioration in his condition necessitated a surgical intervention, and he was scheduled for an elective Ross procedure in May 2022. Upon admission for the procedure, the patient reported experiencing exertional dyspnea classified as New York Heart Association (NYHA) class 2. A cardiovascular examination revealed a wide pulse pressure and a grade II-III diastolic murmur at the left sternal border. Prior to the procedure, the most recent chest X-ray images showed bilateral clear lung fields, with no signs of consolidation, pneumothorax, or pleural effusion (Figure 1). Additionally, echocardiogram views confirmed the presence of a dysplastic bicuspid aortic valve, severe aortic valve insufficiency with reversed flow at the abdominal aorta, myxomatous and significantly prolapsing mitral valve with both anterior and posterior leaflets exhibiting mild mitral valve insufficiency, and mild left ventricular dilation with normal left ventricular systolic function (Videos 1-3). This comprehensive assessment provided crucial information for planning the patient's upcoming elective Ross procedure. Based on the existing records, the patient underwent a Ross procedure through a median sternotomy incision. The procedure involved the transection of the aorta, excision of the aortic valve, mobilization of the coronary buttons, and extraction of the pulmonary autograft. Subsequently, the neo-aortic valve was implanted, and the coronary buttons were reattached. He had a smooth intra-operative course, and an intraoperative trans-esophageal echocardiography (TEE) revealed a severe aortic valve insufficiency (Figure 2), which was promptly repaired during the procedure, resulting in good myocardial function with no significant residual lesions.

**Video 1:** Severe Aortic Regurgitation

**Video 2:** Mitral Valve Prolapse

**Video 3:** Aortic Valve (multiple jets)

**Figure 3:**
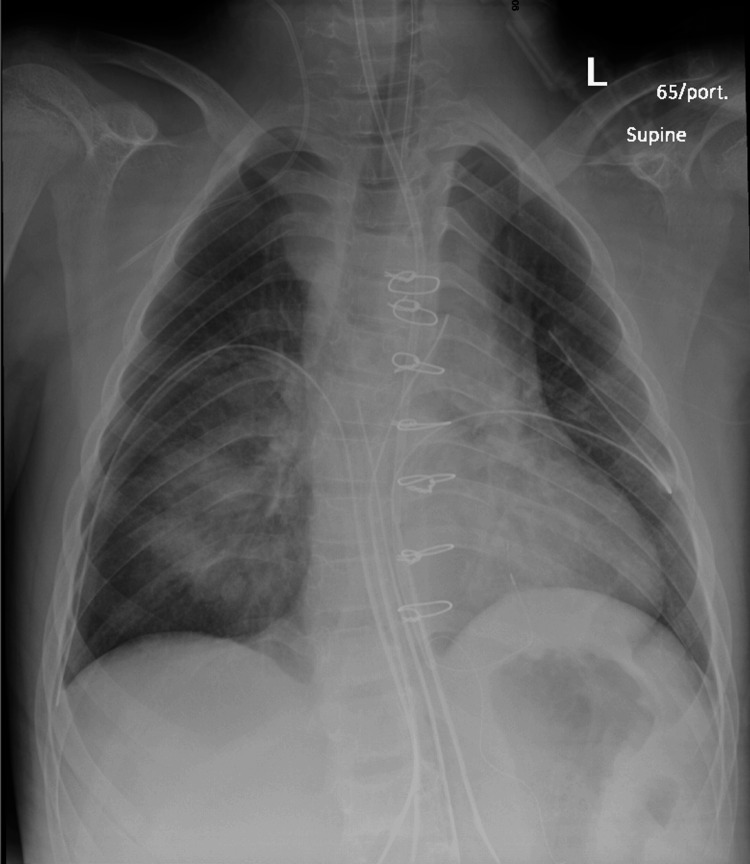
Post-op Chest X-ray

On the second day post-procedure, seven intact midline sternotomy wires and an epicardial pacing wire, along with interval development of bilateral medial upper lobes and lower lung zone airspace opacities were noted (Figure 3). The patient was transferred to the Intensive Care Unit in a stable condition afterwards and was successfully extubated on the following day without any complications. However, on the seventh post-operative day, the patient experienced recurrent spikes of high-grade fever, prompting the initiation of empirical antibiotics and the involvement of infectious disease (ID) team. A work-up of urine culture, blood culture, Brucella abortus, Brucella melitensis, nasopharyngeal aspirate, and Q fever all yielded negative results. A chest computed tomography (CT) revealed a suprasternal collection with pre-sternal and retrosternal extension. In response, the ID team recommended a two-week course of vancomycin (10 mg/kg) and meropenem (20 mg/kg) and advised a repeat chest CT after this period. Subsequent CT scans indicated a moderate dehiscence of the sternotomy at the manubrium, potentially associated with the local infection. As a result, the antibiotic course was extended for an additional two weeks, and another chest CT was scheduled after this period, which showed a reduction in the size of the collection, necessitating an additional two weeks of antibiotics. Following consultation with the relevant cardiac surgery team, it was determined to discharge the patient and closely monitor their progress in the outpatient department. The ID team recommended a two-week course of oral ciprofloxacin and clindamycin upon discharge. A month later, the patient was discharged in a stable condition and prescribed angiotensin-converting enzyme inhibitors (ACEI) and aspirin. The last echocardiography before discharge revealed no obstruction in the left ventricular outflow tract (LVOT), trace neo-aortic valve regurgitation, unobstructed right ventricle-pulmonary artery (RV-PA) conduit, mild conduit valve regurgitation, mitral valve prolapse with mild regurgitation, mild tricuspid valve regurgitation, unobstructed PA branches, unobstructed aortic arch, no echo evidence of vegetations, mild reduction in left ventricular systolic function, and no pericardial effusion (Video 4).

**Video 4:** Post-op: Aortic Valve (short axis)

A year later, the patient was assessed in the outpatient clinic and was found to be clinically stable while taking a daily 81 mg aspirin tablet and a 2.5 mg ACEI tablet (lisinopril). The echocardiogram done then revealed normal aortic valve velocity, mild aortic valve insufficiency, normal pulmonary artery conduit gradient, mild pulmonary artery conduit insufficiency, no stenosis in the right or left pulmonary artery, thickened prolapsed mitral valve leaflets, moderate mitral valve insufficiency, dilated left ventricle, and preserved left ventricular function (Videos 5, 6).

**Video 5:** Last Echo: Aortic Valve (parasternal view)

**Video 6:** Last Echo: Mitral Valve (parasternal view)

## Discussion

Baraitser-Winter syndrome is a complex genetic disorder characterized by a distinct facial appearance, intellectual disability, developmental delays, seizures, and often concurrent cardiac and urinary tract malformations [8]. Managing this condition poses significant challenges, often requiring a comprehensive and multidisciplinary approach, and in certain instances, surgical intervention becomes a pivotal consideration. In our patient's case, they had a congenital bicuspid aortic valve with severe insufficiency and had undergone an extended period of medical treatment to address the associated symptoms. However, as the symptoms persisted, surgical intervention became imperative.

A crucial decision had to be made regarding the choice between mechanical aortic valve replacement and the Ross procedure. The specific challenge stemmed from the unique circumstances presented by the young patient. Mechanical aortic valve replacement carries a heightened cumulative lifetime susceptibility to prosthesis-related complications, presenting a considerable management challenge. Furthermore, the use of mechanical valve necessitates lifelong anticoagulation, which poses a risk of bleeding and thromboembolic events [10].

It is our understanding that Ross procedure was preferred over Ozaki procedure in this case due to its current status as the gold standard surgical approach for severe aortic valve dysfunction in pediatric population and its established practice at our institution [11]. Ross procedure encompasses the replacement of the aortic valve with a pulmonary autograft and the positioning of a homograft in the pulmonary location. Beyond offering relief from the necessity of anticoagulation, this procedure stands as the exclusive aortic valve replacement approach affording sustained long-term viability of the aortic valve substitute [12]. This unique attribute allows for adaptive remodeling and a hemodynamic profile similar to that of the native aortic valve, thus intricately aligning with the complex clinical demands presented by the patient’s condition. Ozaki procedure, on the other hand, aims to address aortic valve disease by reconstructing the patient's native valve using the autologous pericardium to decrease the risk of inflammation and rejection [13]. It represents a novel approach and a less widely adopted method for aortic valve reconstruction, further research and long-term follow-up studies are needed to fully evaluate its effectiveness, durability, and applicability in different patient populations, particularly in pediatric patients [11].

## Conclusions

Baraitser-Winter syndrome is a rare genetic disorder that is caused by mutations in the ACTB and ACTG1 genes. The syndrome is characterized by a wide range of clinical features, including dysmorphic craniofacial features, brain malformation, intellectual disability, and congenital cardiovascular anomalies. In this report, a case of BRWS with congenital bicuspid aortic valve with severe aortic valve insufficiency underwent Ross procedure and is currently in stable condition.
